# A Parts Detection Network for Switch Machine Parts in Complex Rail Transit Scenarios

**DOI:** 10.3390/s25113287

**Published:** 2025-05-23

**Authors:** Jiu Yong, Jianwu Dang, Wenxuan Deng

**Affiliations:** The School of Electronic and Information Engineering, Lanzhou Jiaotong Univeristy, Lanzhou 730070, China; dangjw@mail.lzjtu.cn (J.D.); 13139360529@126.com (W.D.)

**Keywords:** rail transit, object detection, MobileNetV3, convolutional neural network, ResAM

## Abstract

The rail transit switch machine ensures the safe turning and operation of trains on the track by switching switch positions, locking switch rails, and reflecting switch status in real time. However, in the detection of complex rail transit switch machine parts such as augmented reality and automatic inspection, existing algorithms have problems such as insufficient feature extraction, large computational complexity, and high demand for hardware resources. This article proposes a complex scene rail transit switch machine parts detection network YOLO-SMPDNet (YOLO-based Switch Machine Parts Detecting Network). The YOLOv8s backbone network is improved, and the number of network parameters are reduced by introducing MobileNetV3. Then a parameter-free attention-enhanced ResAM module is designed, which forms a lightweight detection network with the improved network, improving detection efficiency. Finally, Focal IoU Loss is introduced to more accurately define the scale information of the prediction box, alleviate the problem of imbalanced positive and negative samples, and improve the relative ambiguity of CIoU Loss in YOLOv8s on the definition of aspect ratio. By validating the performance of YOLO-SMPDNet on a self-made dataset of rail transit switch machines, the results show that YOLO-SMPDNet can significantly improve detection accuracy and real-time performance and has robust comprehensive detection capabilities for rail transit switch machine parts and good practical application performance.

## 1. Introduction

The switch machine is crucial in the switch conversion equipment of rail transit, used for reversing and changing tracks of trains, and plays an important role in the safety of train operation. Parts failure of the switch machine can cause train delays, seriously affecting the normal operation of the train. The traditional fault maintenance methods mainly rely on visual inspection and experience judgment by maintenance personnel for manual inspection and maintenance, requiring a large amount of accumulated experience. The cost of manual inspection is high, the real-time performance is poor, the fault detection efficiency is low, and it lacks robustness to changes in feature diversity. Therefore, the traditional fault maintenance can no longer meet the needs of today’s maintenance and detection. With the increasing popularity of augmented reality technology, virtual objects can be overlaid onto the real maintenance environment and visualized by detecting and locating objects in real maintenance scenarios and tracking and registering them, which can greatly improve the maintenance efficiency of maintenance personnel and reduce maintenance costs [1].

It is crucial to achieve efficient augmented reality fault repair and precise detection of switch machine parts, and the accuracy of detection will directly affect the efficiency of fault repair. With the rapid development of deep learning technology, methods based on deep learning have better robustness in situations such as large differences in part size, simple texture, and lighting changes [2]. Researchers have begun to apply them to parts detection, and single-stage detection methods have faster detection speed and lower equipment requirements and are easy to deploy in industrial scenarios [3,4]. Therefore, in order to achieve more accurate detection of switch machine parts, this article proposes a switch machine parts detection network YOLO-SMPDNet (a YOLO-based Switch Machine Parts Detection Network) based on the YOLOv8s algorithm, taking into account the distribution characteristics of internal parts in complex scene rail transit switch machines. By introducing a lightweight backbone network, designing a lightweight convolution module without parameter attention enhancement, and improving the loss function, the network improves the real-time performance and robustness of detection, adapts to complex environments, and enhances the accuracy and precision of object detection. The main contribution of this work is as follows:

(1) Aiming at the problems of low detection accuracy and missed detection in traditional detection of rail transit switch machine parts, a complex scene rail transit switch machine Parts Detection network YOLO-SMPDNet based on YOLO is proposed, which can effectively improve the detection accuracy of switch machine parts.
(2) YOLO-SMPDNet introduces MobileNetV3 to improve the YOLOv8s backbone network, further reducing the number of network parameters, and designs a parameter-free attention-enhanced ResAM module. After introducing the SPPF front and four Concat feature layers of the network, it forms a lightweight detection network together with the MobileNetV3 network to enhance the representation ability of the detection network.
(3) Due to the relatively vague definition of aspect ratio in CIoU Loss of YOLOv8s, YOLO-SMPDNet introduces Focal IoU Loss to more accurately define the scale information of the prediction box, alleviate the problem of imbalanced positive and negative samples, accelerate network convergence, and improve regression accuracy.
(4) This work further validates the practical application performance of YOLO-SMPDNet by incorporating it into the SLAM system and running it as an independent parallel thread. The results show that YOLO-SMPDNet has a more significant improvement in accuracy for camera mobile shooting compared with static shooting, has a smaller absolute trajectory error and better real-time performance, and further validates the generalization and robustness of YOLO-SMPDNet.

## 2. Related Work

Object detection has significant value in fields such as computer vision, augmented reality, and equipment manufacturing, attracting extensive research from scholars both domestically and internationally. At present, mainstream object detection techniques are mainly divided into two categories: one is traditional methods based on image features, and the other is methods based on deep learning. Traditional methods typically require manual design of feature extractors to obtain feature information of the target, followed by classification using machine learning algorithms such as support vector machines. However, traditional methods based on image features heavily rely on image feature information and have poor robustness in complex object detection scenarios such as large differences in target size and single texture. In addition, the feature extraction process requires a large amount of computation and high hardware requirements, making it unsuitable for mobile scenarios. With the rapid development of deep learning technology, researchers have begun to apply it to object detection. For example, Pechenin et al. [5] trained multiple convolutional neural networks and matched them with detection target data information to achieve accurate detection of targets. However, this method has poor real-time performance and is time-consuming. Therefore, compared with traditional image feature-based object detection methods, deep learning-based object detection methods have better robustness and detection accuracy in scenarios with single textures and large differences in object size.

### 2.1. Two-Stage Object Detection Method

The traditional object detection method process includes region generation, feature extraction, classification, and regression. Region of interest (RoI) generation refers to generating multiple candidate regions in an input image. Usually, methods based on sliding windows or selective search are used for implementation. Sliding windows generate candidate regions by sliding fixed sized windows at different scales and aspect ratios on the image, while selective search combines regions based on low-level features of the image to generate candidate regions.

Feature extraction refers to extracting information from each candidate region that can represent the target features. For the target that needs to be detected, feature extraction usually relies on manually designed feature descriptors. These feature descriptors can capture visual information such as edges and textures of the target, preparing for subsequent classifier classification. Classification and regression classify each candidate region (to determine if it contains the target) and regress (to adjust the position and size of the bounding box). The classification task uses a classifier to classify feature vectors. The regression task uses a regression model (such as linear regression) to adjust the position and size of the bounding box to more accurately locate the target.

Due to the large amount of redundant areas, high computational complexity, and complexity generated by traditional object detection methods, a two-stage object detection method based on deep learning has emerged. It is divided into two steps: first generate candidate regions, and then classify and regress the candidate regions. It has strong targeting and can reduce the range of regional selection. In 2014, Ross Girshick et al. [6] proposed a two-stage object detection method based on region recommendation. This method first extracts candidate regions and then uses CNN to extract and classify the features of the candidate regions to complete the detection task. On this basis, improved two-stage object detection methods such as R-CNN [7] and Fast R-CNN [8] have emerged. R-CNN (region-based CNN) first uses the selective search algorithm to generate candidate regions, then uses CNN to extract features from the candidate regions, and finally uses a support vector machine (SVM) classifier to classify the candidate regions. The introduction of deep learning significantly improves the accuracy of object detection, but its computational efficiency is relatively low. Fast R-CNN is an improvement on R-CNN by introducing ROI pooling layers and directly using CNN to obtain feature maps of images, significantly reducing computational complexity. However, it still relies on selective search algorithms to generate candidate regions, which has a high computational complexity and is not suitable for deployment on mobile devices. Faster R-CNN further improves Fast R-CNN by proposing a region proposal network (RPN) [9] to replace selective search algorithms, significantly improving detection accuracy and speed, laying an important foundation for the development of object detection.

### 2.2. One-Stage Object Detection Method

Although the two-stage method has high accuracy, its detection speed is slow and cannot meet the requirements of real-time performance. The one-stage object detection method based on deep learning does not require the generation of candidate regions but directly predicts on the image, thus having faster detection speed and being suitable for real-time application scenarios. In 2016, Joseph et al. [10] proposed the YOLO algorithm (You Only Look Once), which divides the input image into S × S grids, each responsible for predicting a certain number of bounding boxes and corresponding category probabilities. It can directly output the target category and location coordinates, but the detection accuracy is low, and there are still certain limitations.

In response to the low detection accuracy of the YOLO algorithm, in 2018, the YOLOv3 algorithm [11] integrated multiple scale features and introduced residual modules in the feature extraction network to improve the network’s feature extraction capability. In 2020, the YOLOv4 algorithm [12] improved the feature extraction network to CSP Darknet-53 and added a spatial pyramid pooling layer to expand the receptive field, greatly reducing the performance requirements of the algorithm on hardware devices. In the same year, Ultralytics released the YOLOv5 [13] algorithm, further improving detection speed. In 2023, the Ultralytics team proposed the YOLOv8 [13] algorithm, which further optimized YOLOv5 by using a C2f structure with richer gradient flow. In the head section, the previous coupling head structure was replaced with the current decoupling head structure, separating the classification and detection heads. At the same time, Anchor Based was replaced with Anchor Free, abandoning the traditional anchor box design and directly predicting the center point and bounding box size of the target, simplifying the detection process and improving the model’s detection performance in small targets and dense scenes.

As a lightweight member of the YOLO series, YOLOv8s has demonstrated significant advantages in balancing real-time performance and accuracy, model efficiency, and deployment flexibility [14]. Compared with YOLOv9 [15], it achieves faster inference speed on GPUs through CSPNet and dynamic input resolution optimization, making it particularly suitable for real-time detection scenarios on mobile or edge devices. Although YOLOv9 introduces Transformer to improve the accuracy of small object detection, the increased computational cost leads to an increase in latency. Compared with YOLOv11 [16], YOLOv8s maintains similar accuracy while deploying more efficiently on embedded devices with lower parameter and computational complexity. Additionally, it has a significant advantage in GPU inference speed, making it suitable for high-throughput demand scenarios. In addition, compared with the DETR algorithm based on Transformer [17], YOLOv8s does not require complex post-processing and achieves faster convergence speed and end-to-end detection through anchor optimization and feature pyramid network. It has higher practicality in real-time key fields such as industrial inspection and autonomous driving. Therefore, considering the limited mobile resources such as augmented reality operation and maintenance of rail transit and taking into account factors such as model size and accuracy, this paper will use the YOLOv8s method based on single-stage object detection to detect switch machine parts.

## 3. YOLO-SMPDNet

In the application scenario of point machine operation and maintenance, there are often problems of mixed targets and complex backgrounds in the background of part recognition, which can lead to insufficient feature extraction and low detection accuracy of parts. To this end, YOLO-SMPDNet adopts the MobileNetV3 network [18] as the backbone network to extract features and reduce the number of network parameters and designs a parameter-free attention-enhanced ResAM module to form a lightweight detection network together with the MobileNetV3 network, enhancing the representation ability of the detection network and quickly detecting dynamic objects. Finally, the Focal IoU loss function is introduced to more accurately define the scale information of the prediction box and improve the localization effect.

### 3.1. Feature Extraction Based on the MobileNetV3 Network

The existing deep learning-based object detection methods have disadvantages such as large convolutional kernels, a large number of convolutional layers, and high hardware resource requirements, which result in high parameter count and computational complexity, making them difficult to apply to resource-constrained mobile devices. The MobileNetV3 lightweight network consists of multiple Bottleneck modules. This module is divided into four parts: an inverse residual structure, depthwise separable convolution, an SE (Squeeze and Excitation) module, and an h-swift activation function. First, the dimensionality of the feature representation is increased by using 1 × 1 convolution and residual connections between the input and output. Second, separable convolutions with a depth of 3 × 3 can reduce the number of computational parameters in the network. Finally, feature reuse is achieved through 1 × 1 convolution dimensionality reduction. On the basis of MobileNetV2, it optimizes the redundant part of the network and introduces an SENet attention mechanism, designs an h-swift activation function, etc. The h-swift activation function is

(1) $$h\text{-}\mathrm{swift}[x]=x\frac{ReLU6(x+3)}{6}$$

**Figure 1:**
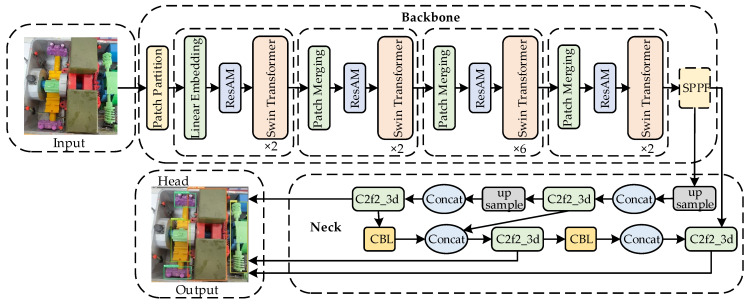
YOLO-SMPDNet structure.

Considering both accuracy and speed, lightweighting is mainly aimed at improving the Backbone part. YOLO-SMPDNet improves the Backbone of YOLOv8s using 15 layers of lightweight MobileNetV3 and adds ResAM modules before SPPF (Spatial Pyramid Pooling Fast) and after four Concat feature layers. While ensuring detection accuracy, shorten the running time to achieve optimal model performance as much as possible. The overall structure of YOLO-SMPDNet is shown in Figure 1.

### 3.2. Lightweight Module ResAM Based on Parameter-Free Attention Enhancement

Due to the significant decrease in the overall computational complexity of the lightweight network compared with the original network, it can lead to a decrease in detection accuracy. YOLO-SMPDNet designs a parameter-free attention-enhanced ResAM module to enhance the representation ability of detection targets and reduce the interference of redundant information. SimAM, with its parameter-free, multi-dimensional attention integration, efficient inference speed, and strong noise suppression ability, has become a better choice than SE, CBAM, and ECA in tasks that require lightweight, real-time performance and robustness to complex scenes.

**Figure 2:**
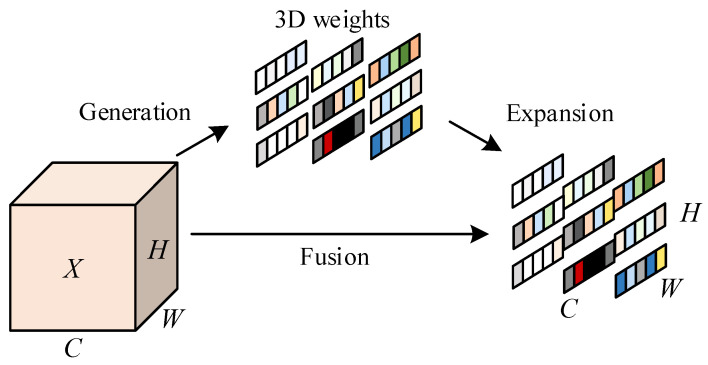
SimAM attention module schematic.

First, the input features are upscaled through a 1 × 1 convolution to aggregate information from different channels. Then, feature extraction is performed through MBConv 3 × 3 convolution with a stride of 2, which combines two key concepts: inverse residual structure and lightweight depthwise separable convolution. The inverse residual structure increases the dimensionality of feature representation through nonlinear transformation, which helps to learn more effective feature representations. Lightweight depthwise separable convolution decomposes the standard convolution into depthwise convolution and pointwise convolution. Depthwise convolution convolves each channel of the input features separately to extract spatial features and outputs the same number of channels as the input. Pointwise convolution uses a 1 × 1 convolution kernel to combine the output channels of deep convolution, adjust the number of channels, and achieve efficient and lightweight design, reducing computational costs and improving the model’s expressive power. Then, through the parameter-free attention SimAM module (Self-improving Attention Mechanism) [19], unlike existing channel/spatial attention modules, SimAM can capture global contextual information and evaluate the importance of each position in the feature map through an energy function, thereby enhancing the model’s ability to focus on key features. It does not require additional parameters to derive 3D attention weights for the feature map, reducing the complexity and computational overhead of the model. It can adaptively enhance key features and suppress redundant information, significantly improving model performance in parts detection while maintaining high computational efficiency. The schematic diagram of the SimAM attention module is shown in Figure 2.

To better achieve attention, it is necessary to evaluate the importance of each neuron. Activating neurons typically suppresses surrounding neurons, known as spatial inhibition. The energy function used to evaluate the importance of neurons is as follows:

(2) $$e_{t}^{*}=\frac{4({\hat{\sigma }}^{2}+\lambda )}{{\left(t-\hat{u}\right)}^{2}+2{\hat{\sigma }}^{2}+2\lambda }$$

**Figure 3:**
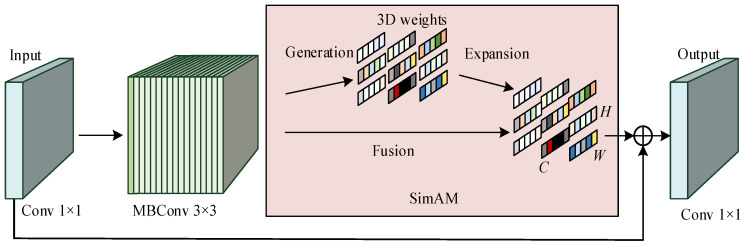
ResAM module structure.

Among them, *t* is the target neuron, *u* and *σ* are the average and variance of all neurons except *t*, and *λ* is the hyperparameter. The lower the energy of *e*, the greater the difference between neuron *t* and the surrounding neurons, and the higher its importance. The importance of neurons is determined by 1/e_t_*. Finally, channel dimensionality reduction is performed through 1 × 1 convolution to eliminate redundant information. Considering that when the network goes deep, in addition to increasing computational resource consumption and model overfitting problems, gradient vanishing and exploding problems may also occur, resulting in the inability to update shallow network parameters. Therefore, residual connection structures are used to optimize the overall training process and improve network performance. The ResAM module structure is shown in Figure 3.

### 3.3. Loss Function of YOLO-SMPDNet

The loss function of the YOLOv8 algorithm is CIoU Loss (Complete Intersection over Union Loss), which not only considers the overlapping area and center point distance of the bounding boxes but also adds the similarity of the aspect ratio to comprehensively measure the difference between the predicted box and the real box. Traditional IoU Loss is insensitive to changes in the scale, aspect ratio, and center point position of bounding boxes. Therefore, DIoU Loss introduces a penalty term for the center point distance based on IoU Loss, which can better reflect the positional differences between two boxes. On the basis of DIoU Loss, CIoU Loss adds a similarity penalty term with the aspect ratio, further optimizing the regression accuracy of the bounding box. The CIoU Loss formula is

(3) $$L_{CIoU}=1-IoU+\frac{\rho^{2}(b,b^{\mathrm{gt}})}{c^{2}}+\alpha \upsilon$$

(4) $$\upsilon =\frac{4}{\pi^{2}}{\left(\arctan\frac{w^{gt}}{h^{gt}}-\arctan\frac{w}{h}\right)}^{2}$$

(5) $$\alpha =\frac{\upsilon }{(1-IoU)+\upsilon }$$

Among them, *ρ* represents the Euclidean distance between two points, and *c* represents the diagonal length of the minimum bounding box between the predicted box and the real box. υ is used to calculate the aspect ratio consistency of two boxes, measured by the tan angle value. *α* is the balance coefficient and is given priority based on the set IoU threshold. When 0 < IoU < 0.5, the coefficient of *α* is 0. When IoU ≥ 0.5, the larger the IoU threshold, the larger the coefficient of *α*.

However, the definition of the aspect ratio in CIoU Loss is relatively vague. When dealing with targets with extreme aspect ratios, the aspect ratio penalty term of CIoU Loss can lead to optimization difficulties, and the weight of CIoU Loss for all samples is the same, making it difficult to distinguish between difficult and easy samples. This can cause the model to focus on simple samples and ignore difficult samples. Therefore, this article introduces Focal IoU Loss [20], where EIoU Loss (Efficient IoU Loss) further optimizes the penalty term for aspect ratio based on CIoU, calculates the differences in width and height separately, and adjusts the prediction box more accurately. At the same time, Focal Loss can reduce the weight of simple samples by introducing modulation factors, making the model more focused on difficult samples and thus improving model performance.

Focal EIoU Loss combines the advantages of Focal Loss and EIoU Loss, which can better handle difficult and easy samples, improve the accuracy of bounding box regression, and better handle overlapping and occlusion problems between targets in dense object detection tasks such as rail transit scenes. The formula for EIoU Loss is

(6) $$L_{\mathrm{EIoU}}=1-\mathrm{IoU}+\frac{\rho^{2}(b,b^{gt})}{{\left(w^{c}\right)}^{2}+{\left(h^{c}\right)}^{2}}+\frac{\rho^{2}(w,w^{gt})}{{\left(w^{c}\right)}^{2}}+\frac{\rho^{2}(h,h^{gt})}{{\left(h^{c}\right)}^{2}}$$

Among them, *hc* is the height of the minimum bounding rectangle between the predicted bounding box and the real bounding box, and *wc* is the width of the minimum bounding rectangle between the predicted bounding box and the real bounding box. The formula for Focal IoU Loss is

(7) $$L_{\left(Focal-\mathrm{EIoU}\right)}={\mathrm{IoU}}^{\gamma }L_{\mathrm{EIoU}}$$

Among them, the hyperparameter *γ* is used to suppress negative samples, and the larger the coefficient of *γ*, the higher the performance of the loss function.

## 4. Experiment and Result Analysis

The development platform configuration set up for this experiment was as follows: the CPU was a 4-core Intel (R) Corporation 12th Gen Intel (R) Core i7-12700H 2.30GHz; the GPU was an NVIDIA GeForce RTX 3070Ti Laptop GPU; the operating system was Windows 11; the language was Python 3.8, conducted on PyCharm 2023; and the deep learning framework used Python 1.8.1. Training, validation, and testing were conducted under the same hyperparameters, with a training epoch of 300, an initial learning rate of 0.01, and a batch size of 4. And comparative experiments were conducted with the current mainstream single-stage object detection methods SSD, YOLOv5, YOLOv8n, and YOLOv8x.

### 4.1. Dataset

**Figure 4:**
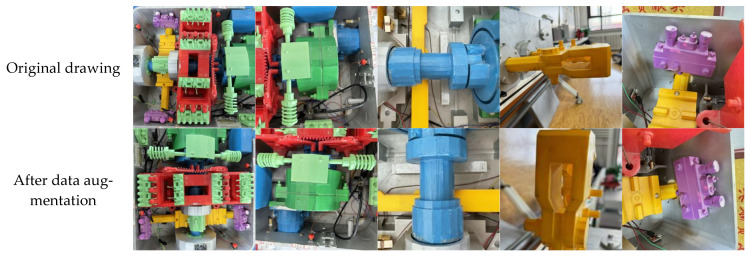
Partial sample images.

**Figure 5:**
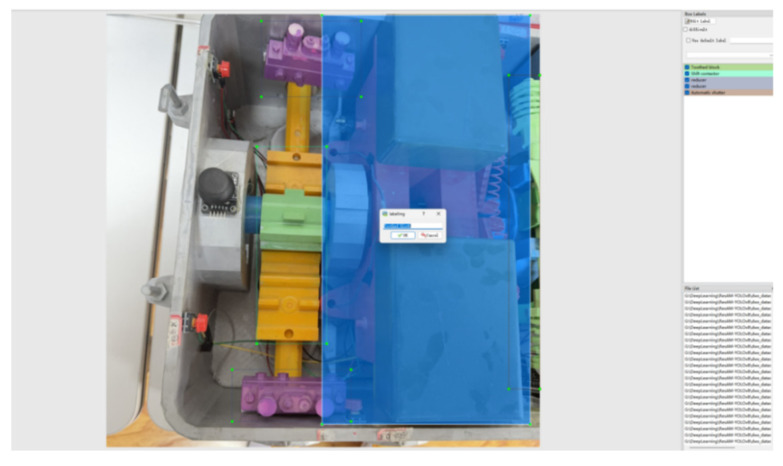
Use labelImg to label each part.

In order to verify the effectiveness of the algorithm, this paper self-made images of ZD6 DC electric switch machine parts, including 2000 images of 8 types of targets. Then, through flipping, rotating, cropping, and scaling operations, a total of 10,523 images were obtained, including 1310 displacement contactors, 1325 automatic switches, 1500 reducers, 1236 spindles, 1200 indicator rods, 1200 operating rods, 1500 motors, and 1252 rack blocks. Some sample images are shown in Figure 4. Finally, the visible parts of the eight types of parts were labeled using labelImg v1.8.1 software, as shown in Figure 5. The labeling criteria were as follows:

(1) Label the visible parts of the eight types of parts with tangents closely attached to the edges.
(2) If a part of the annotated target is obscured, its shape needs to be fully supplemented before annotation.
(3) Images that are blurry, too dark, or overexposed are not framed.
(4) Each target object needs to be individually framed.
(5) Ensure that the box coordinates are not on the image boundary to prevent out of bounds errors when loading data.
(6) For small targets, as long as they can be distinguished by human eyes, they should be marked with frames.

After the annotation was completed, corresponding labels were created, and various types of images were randomly allocated in a ratio of 7:1.5:1.5 as the training, validation, and testing sets for the network.

### 4.2. Ablation Experiment

To quantitatively analyze the detection performance of YOLO-SMPDNet, this paper used YOLOv8s as the benchmark model and conducted ablation experiments on mainstream backbone networks, attention modules in ResAM modules, and loss functions. The experimental comparison of YOLOv8s, YOLOv8x, and the improvement of MobileNetV3 lightweight network on the basis of the original YOLOv8s, as well as the changes in recall and mAP50 with and without ResAM, is shown in Table 1 as the results of the main network ablation experiment.

According to the experimental results, adding the ResAM module resulted in a 0.9% increase in recall and a 2.7% increase in mAP50 compared with the original YOLOv8s network. Compared with the YOLOv8x network, recall increased by 1.2%, and mAP50 increased by 2.1%. Compared with the original YOLOv8s network, the introduction of MobileNetV3 resulted in a 0.2% increase in recall and a 0.1% increase in mAP50. Compared with the YOLOv8x network, recall increased by 0.5%, and mAP50 decreased by 0.5%. This was because the lightweight network significantly reduced the number of parameters and computational complexity, leading to a decrease in network detection accuracy. Therefore, adding a parameter-free attention-enhanced ResAM module can improve detection accuracy with minimal parameter increase; After constructing a new feature extraction network using MobileNetV3 and ResAM, the recall and mAP50 values were higher than YOLOv8s and YOLOv8x, increasing by 3.4% and 6.8% and 3.7% and 6.2%, respectively, verifying the superiority of the proposed network.

In order to analyze the superiority of the proposed ResAM module, the ResAM module was added to YOLOv8-MobileNetV3, and experimental analysis was conducted on other mainstream attention modules, SE [21] and CBAM [22]. The experimental results of the ResAM module are shown in Table 2.

According to the experimental results, compared with the YOLOv8-MobileNetV3 network, adding SE and CBAM to ResAM increased mAP50 by 5.7% and 6.2%, mAP50-95 by 2.0% and 2.8%, and the parameter count by 8.0 M and 6.8 M, respectively. After adding the SimAM attention module, compared with the YOLOv8-MobileNetV3 network, mAP50 increased by 6.7%, mAP50-95 increased by 4.6%, and the parameter count increased by 0.8 M. It could be seen that ResAM, which includes the SimAM attention module, significantly improved the mAP50 and mAP50-95 indicators compared with mainstream attention modules such as SE and CBAM and had smaller parameter counts and better accuracy. The results showed that the ResAM SimAM module effectively improved the performance of the model and was superior to other mainstream attention modules.

In order to analyze and verify the effectiveness of the Focal IoU loss function, the CIoU, EIoU, and Focal IoU loss functions were compared based on YOLO MobileNetV3 ResAM. The experimental results of the loss function are shown in Table 3.

According to the experimental results in Table 3, compared with the original CIoU loss function, the Focal EIOU loss function of YOLO-SMPDNet in this paper had a 0.3% increase in recall rate R and a 0.3% increase in mAP50, with higher accuracy than the two mainstream loss functions CIoU and EIoU. This verified the effectiveness of the Focal EIOU loss function in improving accuracy.

### 4.3. Comparative Experimental Analysis

**Figure 6:**
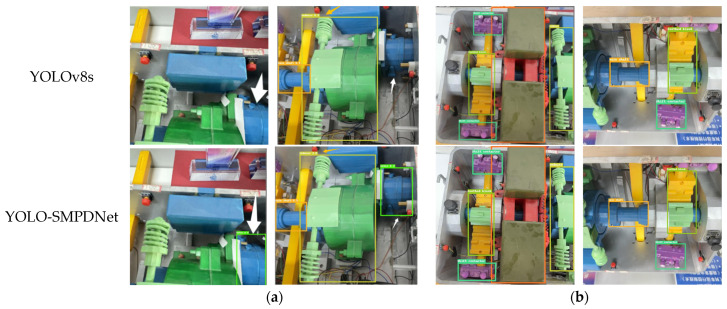
Visualization results of comparative experiments. (**a**) Comparison of motor detection diagrams. (**b**) Comparison chart of prediction boxes.

This article selected the YOLOv8s algorithm and YOLO-SMPDNet to test part images, using YOLOv8s as the benchmark model, and selected representative images of each category in the test set for qualitative analysis. The comparison of detection effects is shown in Figure 6, where the first line represents the detection results of YOLOv8s, and the second line represents the detection results of YOLO-SMPDNet.

As shown in Figure 6a, the electric motor indicated by the white arrow in the figure lacked a part of the body. After multiple downsampling operations by the backbone network to extract features, the target detail information was severely lost, resulting in missed detections. In addition, the original algorithm had unclear features due to complex background and screw blocking, leading to missed detections. Due to the use of a new backbone network structure, YOLO-SMPDNet could better partition part information and retain key information. At the same time, the ResAM module effectively restored target detail information, thereby avoiding missed detections and making the yellow prediction box (indicated by the orange arrow) of the original algorithm more accurate. As shown in Figure 6b, the YOLOv8s algorithm had a prediction accuracy of 0.7, while the YOLO-SMPDNet had a prediction accuracy of 0.9, which was more accurate and could better define the scale information of the target, proving the effectiveness of the YOLO-SMPDNet loss function. Compared with the YOLOv8s algorithm, it could better locate the position of targets in the switch machine and suppress the interference of redundant information, resulting in significant improvements in detection accuracy and capability.

In order to verify the better object detection ability of YOLO-SMPDNet compared with other algorithms, comparative experiments were conducted with the current mainstream single-stage object detection methods SSD, YOLOv5, YOLOv8, YOLOv8s, YOLOv8x, and the latest YOLOv11. Using recall and mAP50 as evaluation criteria, the detection results of different object detection methods in 8 categories of displacement contactors, automatic switches, reducers, spindles, indicator rods, operating rods, motors, and rack blocks in a self-made track parts dataset are presented in Table 4. The standard deviation mAP was 50.2 ± 1.5, and the confidence intervals were [48.5, 51.9].

**Figure 7:**
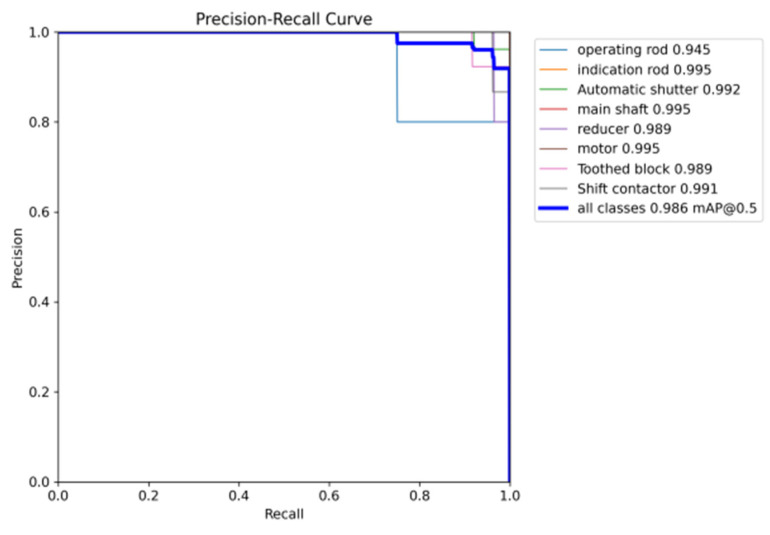
PR curve of YOLO-SMPDNet.

According to the results obtained from Table 4, YOLO-SMPDNet outperformed other single-stage mainstream algorithms in terms of recall and mAP50 values for 8 types of parts. Compared with SSD, YOLOv5, YOLOv8s, YOLOv8x, and YOLOv11, the average recall of 8 types of parts increased by 4.9%, 4.5%, 3.5%, 3.8%, and 0.7%, respectively. The average precision mAP50 values of 8 types of parts increased by 9.0%, 8.5%, 7.1%, 6.5%, and 4.5%, respectively. The experimental results showed that YOLO-SMPDNet had better robustness in situations where there were complex backgrounds, mixed targets, and occlusions in the target of the switch machine. The results showed that compared with the classic algorithms mentioned above, YOLO-SMPDNet significantly improved the detection accuracy of targets and can more accurately locate target parts. Overall, YOLO-SMPDNet had the best target detection ability. The PR curve of YOLO-SMPDNet is shown in Figure 7.

Experimental verification of the performance of the lightweight object detection network and comparison of parameter quantity and GFLOPs with other mainstream single-stage object detection methods were conducted. The comparative experimental results are shown in Table 5. The results showed that the parameter size of YOLO-SMPDNet was only 5.9 M, and the GFLOPs were 16.8. Although GFLOPs were lower than YOLOv11, their other performance was better than other mainstream single-stage object detection methods.

This experiment demonstrated that the YOLO-SMPDNet algorithm had better robustness in situations where there were complex backgrounds, mixed targets, and occlusions in the target of the switch machine. Therefore, compared with the classic algorithms mentioned above, the YOLO-SMPDNet algorithm significantly improved the detection accuracy of targets and could more accurately locate target parts. Overall, YOLO-SMPDNet had the best comprehensive ability in target detection.

### 4.4. Application Performance Analysis

To further validate the practical application performance of YOLO-SMPDNet, the ORB-SLAM3 algorithm [23] was replaced with YOLO-SMPDNet as the object detection module, running independently on parallel threads, and a comparative experimental analysis was conducted. Using the TUM dataset [24] for testing experiments, three sets of high dynamic scene working sequences and three sets of low dynamic scene sitting sequences were selected for system accuracy evaluation. Static represents the camera in an almost stationary state; *xyz* represents the camera moving in the *x*, *y*, and *z* directions; and halfsphere represents the camera’s arc-shaped motion state.

**Figure 8:**
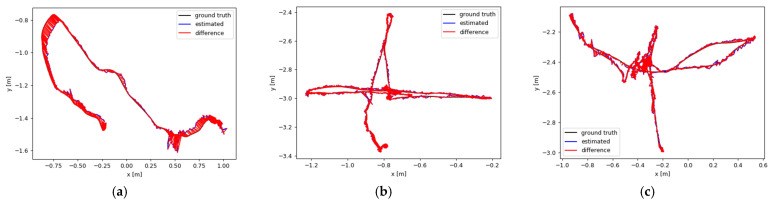

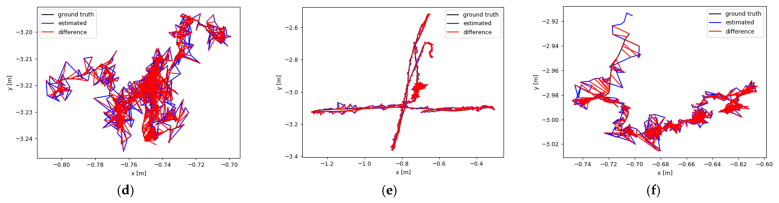
Results of motion trajectory. (**a**) sitting_static, (**b**) sitting_xyz_, (**c**) sitting_halfsphere, (**d**) walking_static, (**e**) walking_xyz, and (**f**) walking_halfsphere.

As shown in Figure 8, the visualization results of the motion trajectory based on YOLO-SMPDNet for static, *xyz*, and half sphere (stationary, moving around the coordinate axis, and traveling in a one meter hemisphere) in the sitting and walking categories are presented. Among them, ground truth represents the true trajectory of camera motion, the blue line represents the true trajectory based on YOLO-SMPDNet, and the red line represents the true trajectory of ORB-SLAM3. It can be seen that YOLO-SMPDNet had smaller trajectory errors and was closer to the real trajectory in the three subsequences of the sitting dataset, with slightly improved performance compared with ORB-SLAM3. Among the three subsequences in the walking dataset, YOLO-SMPDNet was closer to the real trajectory with significantly lower trajectory error than ORB-SLAM3, demonstrating the effectiveness of YOLO-SMPDNet in practical application scenarios.

To further quantitatively analyze the performance of YOLO-SMPDNet compared with ORB-SLAM3, experimental results were compared and tested on six datasets of sitting and walking categories. Using absolute trajectory error ATE for quantitative analysis, its root mean square error RMSE was calculated in meters. The RMSE recorded the error between the true value and the estimated value, and the smaller the value, the closer it was to the true value, indicating that the trajectory of the estimated value was closer to the true value. This was used to evaluate the practical application performance of YOLO-SMPDNet. In addition, the real-time performance of YOLO-SMPDNet was further validated through the average frame rate and the frame time per frame.

As shown in Table 6, in the sitting dataset sequence, it can be seen from different camera poses that the YOLO-SMPDNet algorithm reduced the algorithm error by 44.5% compared with ORB-SLAM3 for stationary camera poses and reduced the algorithm error by 77.3% and 80.6% compared with ORB-SLAM3 for camera poses moving along different trajectories. In the walking dataset sequence, it could be seen from different camera poses that YOLO-SMPDNet reduced the algorithm error by 60.5% compared with ORB-SLAM3 for stationary camera poses and by 95.9% and 94.5% compared with ORB-SLAM3 for camera poses moving along different trajectories. Compared with ORB-SLAM3, YOLO-SMPDNet increased the average frame rate by 8.0% and reduced the frame time by 29.8%, effectively meeting real-time requirements. Therefore, YOLO-SMPDNet had a more significant improvement in accuracy compared with static shooting for camera mobile shooting and had a smaller RMSE for absolute trajectory error, with better accuracy and real-time performance, which also deeply verified the generalization and robustness of YOLO-SMPDNet.

## 5. Discussions

The YOLO-SMPDNet proposed in this article significantly improves the detection performance of rail transit switch machine parts in complex scenarios through multi-module collaborative optimization. The lightweight transformation of YOLOv8s backbone network through MobileNetV3 effectively reduces the number of parameters while maintaining feature extraction capability. This improvement directly alleviates the high dependence of existing algorithms on hardware resources, making them more suitable for edge computing or real-time patrol scenarios. Then, the parameter-free attention-enhanced ResAM module dynamically adjusts channel weights [25], enhancing its feature expression ability for small parts and occluded targets and improving its feature focusing ability in complex backgrounds. Finally, Focal IoU Loss defines the scale and aspect ratio of the prediction box through joint optimization. Compared with CIoU Loss, it reduces the imbalance of positive and negative samples for large-sized parts. These improvements work together to achieve a better balance between accuracy and efficiency in the model.

Compared with the latest mainstream algorithms such as YOLOv9, YOLOv11, and DETR, YOLOv8s has the core advantage of balancing speed and lightweight deployment. Through architecture improvements such as anchor-free box design, dynamic convolution optimization, and C2f module, YOLOv8s achieves millisecond-level fast detection and is a lightweight model while maintaining high detection accuracy. YOLOv8s has more advantages in real-time demanding scenarios such as mobile augmented reality and mobile inspection. In addition, compared with the DETR series that relies on Transformers, YOLOv8s has higher computational complexity and deployment costs [26]. As a single-stage algorithm, YOLOv8s performs better in terms of speed and hardware adaptability. Therefore, YOLOv8s, as the latest member of YOLO series, has the advantage of achieving a better balance between speed and accuracy compared with other mainstream target detection methods. This paper proposes a YOLO-based rail transit switch machine parts detection network YOLO-SMPDNet in complex scenes. Through lightweight design and improved loss function, YOLOv8s shows stronger adaptability in complex rail transit switch machine parts detection scenes while maintaining real-time detection capability [27], significantly improving the detection accuracy in complex rail transit scenes, especially suitable for resource-constrained edge computing scenes.

To further validate the practical application performance of YOLO-SMPDNet, YOLO-SMPDNet was added to the SLAM system. The results showed that YOLO-SMPDNet had a more significant improvement in accuracy for camera moving shooting compared with stationary shooting and had a smaller absolute trajectory error and better real-time performance and further verified the generalization and robustness of YOLO-SMPDNet. Therefore, in the future, multimodal datasets could be constructed to integrate infrared and visible light images to enhance the stability of nighttime detection and to explore temporal information fusion to alleviate the occlusion problem in single frame detection using video stream data. These directions are expected to promote the evolution of the operation and maintenance methods of intelligent mobile augmented reality and automatic inspection in rail transit from “static detection” to “dynamic prediction”.

## 6. Conclusions

To achieve robust detection of rail transit switch machines, traditional object detection methods require many hardware resources, have high computational complexity, and have insufficient feature extraction. This article proposes a YOLO-SMPDNet-based complex scene rail transit point machine parts detection network for complex scene rail transit point machine parts detection scenarios, by introducing the MobileNetV3 network to reduce the number of parameters and solve the problem of low real-time detection performance. A parameter-free attention-enhanced ResAM module was designed, and the Focal IoU loss function was introduced to more accurately define the scale information of the prediction box, solving the problems of insufficient feature extraction and low part detection accuracy. The experimental results demonstrated that the improved algorithm significantly improved the accuracy and speed of part detection and had good practical application performance. However, due to the complex and closed nature of the rail transit scene, the self-made parts detection dataset in this article was relatively limited. The next step will continue to expand the dataset of more equipment parts in rail transit to meet the adaptive detection needs of complex scenarios such as mobile AR and automatic inspection in rail transit.

## Figures and Tables

**Table 1:** Backbone ablation experiment results.

Model	Recall/%	mAP50/%
YOLOv8x	91.3	92.1
YOLOv8s	91.6	91.5
MobileNetV3	91.8	91.6
ResAM	92.5	94.2
MobileNetV3 + ResAM	95.0	98.3

**Table 2:** Experimental results of the ResAM module.

Model	mAP50 (%)	mAP50-95 (%)	Parameter Quantity (M)
YOLOv8s-MobileNetV3	91.6	84.3	5.1
+ResAM-SE	97.3	86.3	13.1
+ResAM-CBAM	97.8	87.1	11.9
+ResAM-SimAM	98.3	88.9	5.9

**Table 3:** Experimental results of loss function (%).

Model	Recall	mAP50	mAP50-95
CIoU	94.8	98.3	88.9
+EIoU	94.9	98.2	88.9
+Focal-EIoU	95.1	98.6	89.2

**Table 4:** Detection results of different object detection methods (unit: %).

Category	SSD		YOLOv5		YOLOv8s		YOLOv8x		YOLOv11		YOLO-SMPDNet	
	Recall	mAP50	Recall	mAP50	Recall	mAP50	Recall	mAP50	Recall	mAP50	Recall	mAP50
Displacement contactor	94.8	91.4	92.2	92.8	93.2	93.3	96.2	94.9	97.3	97.3	96.2	99.1
Automatic shutter	91.9	92.0	92.0	93.6	91.0	92.1	92.0	93.6	91.5	95.1	100	99.2
Reducer	92.4	92.1	91.1	92.8	91.0	91.2	90.1	93.1	95.2	97.9	96.4	98.9
Main shaft	94.6	93.5	94.0	95.5	94.8	95.5	92.3	92.5	94.7	93.4	100	99.5
Indication rod	95.3	91.1	87.5	93.2	95.0	93.2	91.0	92.2	94	95.7	95.1	99.5
Control lever	79.0	78.3	86.3	76.3	85.0	84.8	85.0	84.5	89.7	82.7	75.0	94.5
Motor	83.2	90.8	93.0	94.5	86.5	83.5	85.7	91.8	93.2	94.3	97.9	99.5
Tooth block	90.4	87.6	88.7	82.1	96.3	98.4	98.1	94.2	99.4	96.5	100	98.9
Average value	90.2	89.6	90.6	90.1	91.6	91.5	91.3	92.1	94.4	94.1	95.1	98.6

**Table 5:** Performance comparison of object detection methods.

Model	Parameter Quantity/M	GFLOPs	Real Time/FPS
YOLOv3	68.5	66.7	32
YOLOv5	12.6	18.8	133
YOLOv8	11.2	25.7	123
YOLOv11	7.3	15.1	137
YOLO-SMPDNet	5.9	16.8	142

**Table 6:** Comparison experimental results of absolute trajectory error RMSE and real-time performance.

Sequence	RMSE		Error Reduction Amplitude/%
	ORB-SLAM3	YOLO-SMPDNet	
sitting_static	0.009	0.005	44.5
sitting_xyz	0.044	0.010	77.3
sitting_halfsphere	0.047	0.009	80.6
walking_static	0.015	0.006	60.5
walking_xyz	0.270	0.011	95.9
walking_halfsphere	0.291	0.016	94.5
Average frame rate/fps	49.3	53.6	8.0
Time consumption per frame/ms	27.2	19.1	29.8

## Data Availability

The data are contained within this article.
